# Widening Patient Engagement for Rare Disease Drug Trials: The Perspectives of Patients With Idiopathic Pulmonary Fibrosis on Participating in Clinical Drug Trials and Drug Trial Design

**DOI:** 10.1111/hex.70260

**Published:** 2025-04-15

**Authors:** Julia Frost, Widening Engagement Patient Advisory Group, Jessica Mandizha, Steve Jones, Chantal van den Dungen, Lauren Asare, Catherine Pope

**Affiliations:** ^1^ Health and Community Sciences, University of Exeter Medical School University of Exeter, South Cloisters, St Luke's Campus Exeter UK; ^2^ Royal Devon University Healthcare NHS Foundation Trust, Royal Devon and Exeter Hospital (Wonford) Exeter UK; ^3^ Action for Pulmonary Fibrosis Peterborough UK; ^4^ European Pulmonary Fibrosis Federation Overijse Belgium; ^5^ Chargée des relations internationals, ABFFP association belge francophone contre la fibrose pulmonaire Rebecq Belgium; ^6^ Patient and Public Involvement, NIHR Applied Research Collaboration South West Peninsula (PenARC) University of Exeter, South Cloisters, St Luke's Campus Exeter UK; ^7^ Nuffield Department of Primary Care Health Sciences Radcliffe Primary Care Building, Radcliffe Observatory Quarter Oxford UK

**Keywords:** idiopathic pulmonary fibrosis, market access, patient engagement, qualitative research, randomised controlled trials, rare diseases

## Abstract

**Background:**

Research about patient engagement for people with rare diseases has identified how the experiences of some members of the public are overlooked in relation to clinical trial design and trial participation. As part of a knowledge transfer partnership (KTP), the authors were granted access to patient insight reports about the needs of people with idiopathic pulmonary fibrosis (IPF), to inform clinical trial design and marketing strategy. These were contrasted with data from qualitative interviews, informed by and collected from people with IPF and the clinical staff who recruit them to trials.

**Objective:**

To identify patient and professional perspectives for IPF drug trials to create opportunities for innovation in patient engagement.

**Design:**

Ethnography. Qualitative researcher embedded in a pharmaceutical organisation.

**Setting and Participants:**

International patient insight reports to inform a clinical trial protocol (*n* = 1) and marketing strategy (*n* = 6), including the experiences of over 100 patients with IPF. In the United Kingdom, interviews with patients with IPF (*n* = 32) and the staff who support them clinically and recruit them to trials of new medicines (*n* = 19) at one specialist interstitial lung disease (ILD) centre.

**Results:**

Methodological practices inherent in inpatient insight reports ensured the perspectives of some people with IPF were overlooked. Interviews with a more marginalised population of people with IPF, and the staff who support them, identified that some found trial information confusing, trial practices frustrating and the opportunities to engage in trial design absent.

**Discussion:**

Current pharmaceutical practices of working with contract research organisations and patient organisations exclude the perspectives of patients with IPF who do not engage with either. Trial recruitment information needs to be tailored to the needs of individuals, and trial processes need to enable a wider group of patients to participate.

**Conclusions:**

People with IPF want the opportunity to participate in drug trials and trial science. However, methodological rigour and deliberative practices are required to enable a wider group of patients to have a stake in the design and conduct of drug trials for rare diseases. The challenge now is for regulators to mandate such inclusive practices and for pharmaceutical organisations to adopt them.

**Patient or Public Contribution:**

A Patient Advisory Group (PAG) comprising six people with IPF gave input on the research protocol and then on the scope and content of the ongoing research. Two patients from international patient organisations served as a Steering Group (SG). Members of these groups provided their interpretations of the study findings and gave insight on their experiences in clinical design and participation.

## 1 Introduction

Idiopathic pulmonary fibrosis (IPF) is a chronic, progressive and life‐limiting condition. Globally, the prevalence of IPF ranges from 7 to 1650 per 100,000 persons [1]; with a global incidence rate of 0.2–93.7 per 100,000 per year [2]. Symptoms of IPF include a dry cough, dyspnoea and fatigue, with the severe symptom burden and uncertainty about the degenerative nature of the disease impacting all aspects of quality of life [3]. Research priorities for patients living with IPF include medications to stabilise and ultimately to reverse scarring of the lungs, and medication to improve lung function [4]. Currently, two antifibrotic medications are available (Nintedanib and Pirfenidone), although they are not suitable or effective in some people due to differential patient biology [5]. Until recently, UK guidelines restricted the prescription of Nintedanib until forced vital capacity (FVC: the maximum capacity of air that a patient can exhale after a maximum inspiration) fell to ≤ 80% predicted, although the beneficial effects of antifibrotic medications were thought to occur at higher FVC values [6]. Although the current therapies can reduce disease progression, they can neither stop nor reverse the decline in lung function. Patients with IPF may be able to access new treatments via clinical drug trials, designed to investigate the efficacy of those treatments [2], as well as nondrug trials, such as studies about pulmonary rehabilitation [7].

A review of clinical trials for IPF over 25 years concluded that innovation was only possible due to clinicians, patients, patient organisations and the pharmaceutical industry ‘working together towards a common goal’ [8]. The regulation of patient engagement in drug development is predominantly via the US Food and Drug Administration [9], the European Medicines Agency [10] and International Council for Harmonisation of Technical Requirements for Human Use [11], with the latter currently consulting on how best to incorporate patient perspectives in drug development to “improve the quality, relevance, safety and efficiency of drug development and inform regulatory decision making” [12]. One way in which industry engages with patients is via patient insight gathering, often conducted by third‐party vendors (e.g., contract research organisations [CROs]), with the objective of collating patient perspectives to reduce the patient burden, optimise trial delivery and ultimately inform commercial success [13]. Commentators note that although the procedures for collecting patient insight or ‘patient voice’ reports are mandated but not prescribed, there is an expectation of methodological rigour in their conduct [14, 15].

This research was funded by a UK Research and Innovation/Medical Research Council (UKRI/MRC) Innovation Scholars secondment, the objective of which was to foster knowledge exchange between academia and the biotech industry. J.F. worked with a local patient support group (Exeter Patients in Collaboration for Pulmonary Fibrosis Research EPIC PF), expert patient organisations (Action for Pulmonary Fibrosis; Association Belge Francophone Contre La Fibrose Pulmonaire; European Pulmonary Fibrosis Federation) members of which subsequently constituted the Steering Group for the project; local clinical staff (J.M.); a patient and public facilitator (L.A.); a medical sociologist (C.P.), and a pharmaceutical company (hereafter ‘Biotech’) who had recently discontinued a research programme for IPF [16, 17].

We initially conducted a narrative qualitative synthesis to explore how patients and other members of the public engage with drug development for rare diseases [18]. We searched for articles about orphan drug trials that were indexed with any of four highly discriminating methodological MeSH terms: qualitative research, interviews, focus groups and patient participation. This retrieved a sample of 262 abstracts; however, when we screened the full‐text papers, many did not contain recognised methods of qualitative data collection and analysis. Thus, screening produced a sample of 13 papers, which we analysed using narrative synthesis methods. Our analyses identified that patients and caregivers need to be involved in all aspects of trial design; however, ‘inclusion’ is often suboptimal, because research is typically pharmaceutical or clinician‐led, with patients included as subjects, or included as workshop participants, where only the most engaged patients are present [18]. As none of the included papers in the review were about IPF, and as a corrective, the aim of this paper is to identify patient and professional perspectives for IPF drug trials to create opportunities for innovation in patient engagement.

## 2 Methods

### 2.1 Qualitative Study Design

Ethnography of Biotech's patient engagement workstream pertaining to drug development. Interviews with patients with IPF and staff who support them and recruit them to clinical trials [19].

### 2.2 Sampling

Although seconded to Biotech, J.F. identified seven insight reports. These are commercially sensitive and sometimes confidential products of market research procured by Biotech (conducted by commercial organisations between 2019 and 2020) which concern patient perspectives on having IPF and views about available and potential treatments, and which could inform possible commercialisation of a drug‐in‐progress. Biotech granted permission to analyse these reports as anonymised data sources, and without sharing commercially sensitive information. This afforded the authors the opportunity to contrast pharmaceutical research with empirical health services research, to identify opportunities for innovation.

For the empirical interviews, patients were sampled from both the incidence and prevalence clinic lists of a consultant respiratory physician at Exeter South West Peninsula Interstitial Lung Disease Service. Patients on the prevalence list were more likely to be local to an interstitial lung disease (ILD) centre and to have participated in clinical trials, whereas those on the incidence list were more likely to have been referred from across the [Region] for diagnosis and consideration for antifibrotic therapies. From discussions with members of the multidisciplinary team at Exeter South West Peninsula Interstitial Lung Disease Service, and consideration of the numbers of new patients being diagnosed with IPF across the South West of England, a sample of 30 was considered an appropriate number to be recruited in the timeframe and with the available resources. This would enable the inclusion of patients in receipt of medication and those who were not, and those who had participated in clinical trials of medications, and those who had not, with the aim of maximising our understanding of patients’ experiences.

Clinical staff were sampled for maximum variance [20] from the Exeter South West Peninsula Interstitial Lung Disease Service, where the Consultant Respiratory Physician is based, and included staff involved in all aspects of IPF patient care and research pathways, as well as academic staff involved in the delivery of trials for IPF therapies.

### 2.3 Recruitment

The consultant respiratory physician's caseload was reviewed by a National Institute for Health Research Clinical Research Network (NIHR CRN) research nurse who identified patients who met the sampling criteria (*n* = 130). As above, these were all patients on the incidence and prevalence lists of a participating respiratory physician, to include patients who had taken part in one or more clinical drug trials, and those who had not. The research nurse contacted eligible patients, then sent those interested a participant information sheet and an expression of interest form, which when signed was returned directly to J.F. On receipt of the form, J.F. contacted the potential participant and provided further information. All staff working in the ILD service, and those who referred patients to the service and or recruited patients to clinical trials (*n* = 64) were also contacted by the research nurse. The same recruitment process for patients was followed for the potential clinical and trial staff participants.

### 2.4 Data Collection

Permission to use the patient insight reports, in anonymised form, was granted by Biotech's scientific publication leader. All available patient insight reports were collected and anonymised (hereafter Reports 1–7).

Interview topic guides for patients and staff were developed with the Patient Advisory Group (PAG) [21]. These were topics of importance to patient and caregiver members of the PAG: patient and clinician perspectives about the diagnosis of IPF, available treatments, access and participation in clinical trials of new medicines for IPF and orientations towards patient engagement in clinical trials research. Ethical approval for the project was granted in April 2022. Mindful of the impact of COVID on people with a degenerative respiratory condition, and NHS staff working at capacity, all interviews with patients, clinical and trial staff were conducted remotely either by Teams, Zoom or telephone, as the participant preferred. Interviews were transcribed verbatim by a GDPR complaint transcriber, and with the patient insight reports, anonymised and managed in MAXQDA 2022 software [22]. All participants were remunerated for participation [23].

### 2.5 Data Analysis

J.F. used the Critical Appraisal Skills Programme [24] tool to assess the patient insight reports. Although it is suggested that two people should independently undertake critical appraisal, only J.F. had permission to access the patient insight reports. This was mitigated by J.F. discussing the methodological rigour of the papers, including anonymised extracts, with C.P., an internationally recognised expert on qualitative research [25]. Appraisal of qualitative research is considered contentious because there is limited consensus about what makes a study good [26, 27, 28], and here appraisal was undertaken as a form of familiarisation [29]. The report contents were analysed using discourse analysis, which focuses upon the socio‐cultural and political context in which text and talk are captured [30], with the goal of identifying how the experience of IPF was constructed and curated [31].

Abductive analysis was then used to compare and contrast the form and content of the patient insight reports and interview data, with interpretations discussed regularly with the PAG and wider research team, to check their interpretation of the data set [32]. Empirical data were anonymised and participants given pseudonyms (C designates ‘clinical staff’, T ‘trial staff’ and P ‘patient’).

## 3 Results

Seven historical and international reports relating to clinical trial design and for marketing and commercial purposes, with commercially sensitive information redacted, dating from 2019 to 2020 included qualitative data consisting of interviews with 77 patients with IPF, 15 caregivers, 102 physicians, and observations with 37 patients and 15 caregivers.

Interviews were conducted with 32 people with IPF, and 19 members of staff who support them clinically across community and hospital settings, and those recruit them to trials of new medicines at one specialist ILD centre in the United Kingdom. The interviews were conducted in 2022–2023^1^ and lasted between 20 and 90 min.

### 3.1 Analysis of Patient Insight Reports

Tabulation of the research design, sample and countries included in the patient insight reports demonstrated that much of the data were collected from the global north [33], with the bulk from North America and Europe (Table 1). Although data were said to have been collected via interviews, observations, focus groups or surveys, beyond that little or no methodological detail was provided.

**Table 1:** Patient insight reports: Research design, sample and countries.

Contract research organisation	Country									
	United Kingdom	France	Germany	Italy	Spain	Australia	Brazil	Canada	Japan	United States
Report 1										
Health professionals (survey)		(42)	(50)						(65)	(87)
Report 2										
Patients (ethnography)	**3**	**9**		**10**						**15**
Patients (interviews)	**1**	**4**	**3**		**4**			**3**	**6**	**10**
Caregivers (ethnography)	**0**	**5**		**5**						**4**
Caregivers (interviews)	**1**	**0**	**2**		**3**			**2**	**2**	**5**
Report 3										
Patient (interviews)	**2**	**2**	**7**							**7**
Health professionals (interviews)	**11**	**11**	**11**							**15**
Report 4										
Patients (focus group)										**4** (21)
Caregivers (focus group)										**7** (29)
Report 5										
Health professionals	(10)	(10)	(10)	(10)	(10)	(10)		(10)		(20)
Report 6										
Patients (interviews)	**3**	**3**	**3**	**3**	**3**	**1**	**3**	**3**	**3**	**3**
Health professionals (interviews)	**3**	**3**	**3**	**3**	**3**	**3**	**3**	**3**	**3**	**3**
Report 7										
Health professionals (interviews)	**6**	**6**	**6**		**6**					

*Note:* N in bold = qualitative; N in parentheses = survey. Columns cannot be totalled, as some participants appear under more than one research method.

All available reports were included to ensure representation of methodological practices and the substantive knowledge about IPF that was created (Table 2). This process demonstrated that few reports detailed how patients were accessed, ethical practices or how the data were analysed. Reflecting the needs of industry, the reports were conducted at various phases of the drug development lifecycle. For example, we identified one online ethnography to understand patient perspectives of living with IPF (Report 2) [13], and a Target Product Profile (TPP), the purpose of which is to capture the vision of the product, the needs of the market and enable commercialisation (Report 5) [34].

**Table 2:** Patient insight reports: Critical appraisal (CASP).

	1. Clear statement of aims?	2. Appropriate methodology?	3. Research design appropriate?	4. Recruitment strategy appropriate	5. Data collection address the research issue?	6. Relationship between researcher and participant?	7. Ethical issues taken into consideration?	8. Data analysis sufficiently rigorous?	9. Is there a clear statement of findings?	10. Value of the research
Report 1	X	?	?	?	?	X	X	X	X	?
Report 2	√	√	√	?	√	X	X	√	√	√
Report 3	√	√	√	√	√	X	X	√	√	√
Report 4	√	X	?	?	?	X	X	X	X	?
Report 5	√	?	?	?	X	X	X	X	X	?
Report 6	√	?	√	?	√	X	X	X	X	?
Report 7	√	√	√	√	√	X	X	X	?	√

*Note:* √ = yes; X = no; ? = cannot tell.

When synthesised, the reports form a significant data set of patient and physician perspectives about IPF, focused on diagnosis, treatments and unmet needs (Table 3).

**Table 3:** Patient insight reports: Results.

Theme and data source	Country									
	United Kingdom	France	Germany	Italy	Spain	Australia	Brazil	Canada	Japan	United States
Diagnosis										
First symptoms (patients) Report 6	Patients know IPF by now, yet first symptoms were almost never associated with IPF. Something is not right—So, let us wait a bit. Many roads lead to a pulmonologist. That time is irreversibly lost for patients and their lung function.									
Diagnosis process (patients) Report 6	Pulmonologists take an average of up to 3 months to diagnose IPF. Diagnosing IPF means running many tests. The diagnosis of IPF is a collaborative effort—Strength(s) of the MDT. Most countries rely on the traditional staging model. Negative feelings dominate; however, new treatment options boosted positive feelings. The physician–patient dialogue is considered satisfactory by patients. Overwhelmingly negative feelings are evoked in patients through diagnosis. Patients need more information that they can take home. Patient support groups are a double‐edged sword. There is no typical IPF patient, but most have similar characteristics.									
Quality of life (patients) Report 6	Patients are impacted by IPF every day in all activities. Family members are impacted by IPF too. Financial worries are added on top when the IPF patient is still working. Death and disease progression were ever present for the IPF patient									
Expectations for diagnosis (physicians) Report 6	IPF specialists would like to offer additional support to their IPF patients. Higher awareness of IPF among HCPs and our society would lead to an earlier diagnosis.									
Treatment										
Experience of treatment (patients) Report 1		Over half of the patients with mild IPF are prescribed an antibiotic. Number of patients receiving Nintedanib over Pirfenidone is increasing.							Patients who are currently prescribed treatment visit HCPs twice as often as those not on treatment. About 1/3 of patients with mild IPF are prescribed an antibiotic. Number of patients receiving Nintedanib is increasing.	Patients in United States have double the pre‐diagnostic time of German patients. The majority of patients with mild IPF are prescribed an antibiotic. Number of patients without AF treatment is increasing.
Model of patient post‐diagnosis patient flow (physicians) Reports 2 and 3	Referral to accredited IPF centres of excellence is dictated by UK guidelines. NICE guidelines in the United Kingdom restrict AF to 80%–50% FVC range. Patient choice in treatment varies. Pulmonologists usually invite patients to weigh up treatments. Nurses are formal members of the MDT. BlueTeq platform is required for IPF medication. Has a yearly rehab limitation. Continuation of AFs in the United Kingdom is not common due to NICE restrictions.	Pulmonologists and nurses are particularly committed to educational supplements at diagnosis, with many offices tailoring and printing their own materials to have on hand to explain IPF. Most death‐centric conversations.	Hierarchical order, which limits nurses' role and ability to intervene. Nurses are not allowed in educational or emotional support consultations immediately following diagnosis. Their roles are more rigidly tied to the execution of care. Informational material is rarely used, as pulmonologists consider it distracting and less authoritative than their own direct explanations. There are no notable administrative challenges. A long application process.							AF treatment delays occur by pulmonologists choice. Patient choice in treatment varies. Pulmonologists usually invite patients to weigh up treatments. Private insurance and medicare complexities in the United States drive significant delays and confusion. Getting O_2_ is an incredibly painful process that requires patients to jump through hoops.
		AF treatment delays occur by pulmonologist's choice. Pulmonologists have taken an authoritarian role, presenting their recommendations as directives with little opportunity/encouragement for input.								
Treatment (physicians) Report 6	IPF specialists perceived both AF drugs similarly with regard to efficacy. Discussing the side effect profile with patients is key to deciding which treatment to prescribe. AF drug switches are rare. IPF specialists need to clearly manage patients' expectations.									
Treatment (patients) Report 6	Patients are not as involved in the treatment decision as IPF specialists believe. Around half of the IPF patients were prescribed an antifibrotic at diagnosis. IPF patients are very compliant—IPF specialists support them in managing side effects. IPF patients have clear expectations regarding their AF treatment. Pulmonologists are the main contact, nurses play a minor role, GPs almost none. Costs are an issue in the United States and Brazil, less in other countries. IPF patients face many challenges and obstacles before they receive an antifibrotic. Challenges in receiving an AF drug vary from country to country.									
Disease progression (physicians) Reports 4 and 5		Despite patients being described as stable on AFs they tend to exhibit a gradual FVC decline.							Despite patients being described as stable on AFs they tend to exhibit a gradual FVC decline.	
Unmet needs										
Patient ‘types’ (constructed by CRO) Reports 2 and 3	*Resigned cynic* Acceptance of their ‘lot in life’.	*Measured pragmatist*							*Measured pragmatist*	*Proactive fighter* Remain hopeful and optimistic.
			Reluctant to give up vices that may exacerbate IPF.							
Involvement (caregivers) Reports 2 and 3				Extended family is heavily involved in making sure to support IPF patient in any way they can.					Caregivers only get involved in tactical management of IPF.	
Model of patient unmet needs (constructed by CRO) Reports 2 and 3	Rigid NHS policies that help institutionalise ‘wait and watch’.	Delay in seeking care for fear of reprimand by HCP about smoking.		Especially strong taboos and cultural resistance around O_2_ therapy.					Fundamental adoption/approval barriers to AF treatment.	Perceived delay due to referral‐based health care system. More patient involvement/choice in treatment.
Unmet needs (physicians) Reports 5	Lack of better efficacy drugs, which can modify/cure the disease by reversing the lung damage. Lack of significant reduction in mortality. Lack of better tolerable drugs.	Lack of better efficacy drugs that can cure IPF and not just manage or stabilise the FVC by slowing the decline. Drugs with less frequency of dosage. Lack of early IPF diagnosis and a follow‐up marker to evaluate drug performance. Lack of drugs that can reduce mortality. Lack of drugs that would cause lesser side effects.	Lack of a drug with significantly improved efficacy that can cure IPF and not just stabilise the FVC by slowing the decline Lack of early IPF diagnosis. Lack of drugs with lesser side effects that will eventually improve adherence to medicine. Lack of a drug that shows a marked improvement in the symptoms such as exertional dyspnoea. Drugs with less frequency of dosage.	Lack of treatment choices and better efficacy drugs—Need more choices of products and products that can restore the respiratory function. Drugs with minimal side effects—Few respondents feel that side effects are a major unmet need that they can not neglect.	Cure IPF by reversing the lung damage. Further reduction of FVC decline than what the currently available drugs offer. Clinical trial data showing the efficacy of both antifibrotics when used in combination. Drugs or a combination of drugs that can work on different pathways of lung fibrosis. Clinical trial data that evaluate antifibrotic drugs in patients who are receiving anti‐aggregating agents too.	Lack of better efficacy drugs, which can improve the lung function significantly and not just stabilise the disease/slow the disease progression. Lack of significant reduction in mortality rate. Lack of drugs that can improve quality of life by reducing respiratory distress.		A cure for IPF: Need a drug that can either halt disease progression or actually reverse the progression of fibrosis. Lack of drugs with higher efficacy and minimum side effects: More treatment options with good toxicity profiles. Long‐term stability/efficacy: Need more drugs with long‐term stability characteristics because those patients who go for transplant are the ones taking antifibrotics so clearly these patients were unstable with Pirfenidone and Nintedanib.		Lack of better efficacy drugs—Current SoC can only delay disease progression but nothing to actually cure the disease by reversing the damage. Lack of drugs with minimal side effects and less drug–drug interactions. Lack of awareness among respondents and patients about the disease leading to late diagnosis. Lack of more treatment options. Lack of a standard treatment protocol.
Most important clinical end points (physicians) Report 5	Disease progression (measured by FVC decline and CT scan). Mortality (measured by calculating respiratory mortality). QoL (measured by improvement in breathlessness and cough, SGRQ, 6MWT). Safety and tolerability. Occurrence of events (measured by time to first acute IPF related exacerbations and frequency and decrease in respiratory‐related hospitalisation).	Disease progression (measured by functional and radiological tests). Mortality (measured by all‐cause mortality such as mortality due to cardiac or digestive causes or just respiratory mortality alone). QoL (measured by SGRQ—Though not used practically by most respondents due to its length/complexity), BOD test, reduced coughing, CRQ. Occurrence of events (measured by endpoints such as first respiratory hospitalisation, number of overall respiratory‐related hospitalisations). Safety and tolerability.	Mortality (measured by respiratory mortality). Disease progression (measured by functional tests such as PFT, DLco, 6MWT, treadmill test and radiological tests such as CT scan). QoL (measured by LCQ total score, the VAS cough and urge to cough, K‐BILD, EQ‐5D, BORG scale). Occurrence of events (measured by endpoints such as first respiratory‐related hospitalisation, number of respiratory‐related hospitalisations). Safety and tolerability.	Improvement in QoL (assessed through 6MWT, SF36, MRC, reduction in dyspnoea and mini‐mental score test. Note: SGRQ is perceived as theoretical and not feasible for patients). Disease progression (assessed through decrease in DLCO, level of FVC decline (in mL), diffusion test and decrease in no. of hospitalisations due to exacerbations). Decrease in mortality. Safety and tolerability.	Disease progression (measured by functional and radiological tests). Mortality (measured by all‐cause mortality and especially respiratory‐related mortality). QoL (measured by SGRQ, CAC, ACT, ECOG, RCC dyspnoea scale, BORGs test). Occurrence of events (measured by endpoints such as hepatotoxicity, renal failure, time to first respiratory hospitalisation). Safety and tolerability.	Reduction in disease progression in terms of FVC decline rate, PFT, 6MWT and CT scan outcomes and lower mortality rate.		Stopping disease progression by decreasing the decline of vital functions, especially FVC and DLCO, Improvement in the mortality (both all‐cause mortality and respiratory‐related mortality), increase in QoL tested by St. George score questionnaire and MRC and SF36 tests.		Reduction in mortality rate. Reduced disease progression in terms of FVC decline rate, PFT, 6MWT and CT scan outcomes.
Appeal of a trial (patients and caregivers) Report 4										Appeal of the trial is: Provision of hope when they have been told there is little. Feeling of proactively fighting rather than passively awaiting progression of disease. Knowing that ‘someone’ is working towards better outcomes for them.
Recommendations for study development (patients and caregivers) Report 4										Ensure empathy and understanding… Set clear expectations… Offer control and hope… Access to information and support… Meaningful support services…
Expectations from a novel therapy (physicians) Report 5	Efficacy should be better or at least same as Pirfenidone and Nintedanib with increased tolerability; reduction in mortality, improve QoL, treatment option for mild cases and reduction in respiratory‐related hospital admissions.	Reverse FVC decline or at least reduce disease evolution.	Should reverse FVC decline and cause lesser side effects.	Development of targeted therapies to increase the number of responders; better safety and tolerability.	Should at the least revert lung lesions or at least provide a 20% improvement in the FVC and improve QoL	Should at the least stabilise disease at an affordable price with minimum adverse effects.		More efficacious drugs that can not only prevent the progression of IPF but even reverse it measured by improved forced vital capacity and improvement in the lung function in the CAT scan.		Reverse or at least halt disease progression and positive response in terms of patients' self‐reported symptoms and well‐being.

Abbreviations: 6MWT, six‐minute walking test; ACT, asthma control test; AF, antifibrotics; BILD, brief interstitial lung disease; BORG RPE, Borg rating of perceived exertion; CRO, contract research organisation; CRQ, Chronic Respiratory Disease Questionnaire; EQ5D, EuroQol‐5 dimension; FS36, The 36‐Item Short Form Health Survey; FVC, forced vital capacity; HCP, health care professional; IPF, idiopathic pulmonary fibrosis; LCQ, Leicester Cough Questionnaire; NHS, National Health Service (UK); NICE, National Institute for Health and Care Excellence; PFT, pulmonary function tests; QoL, quality of life; SGRQ: St. George's Respiratory Questionnaire; VAS, visual analogue scale.

We identified how patient and professional experiences and needs get constructed by commercial interests. For example, Report 5 undertook quota sampling to gather the perspectives of ten pulmonologists from each of eight countries (20 from the United States). For those sampled from the United Kingdom, there is no indication as to whether they work in secondary (hospital) or tertiary (specialist) care and whether they have a role in prescribing medicines specifically for IPF. Similarly, in Report 6, three patients were sampled from each of ten countries, with the only inclusion criteria being that patients have IPF for more than 5 years and are younger than 75 years. Thus, in both reports, it is unclear which patients and staff are being represented or why.

In Report 6, the output of the three patient interviews per country is a (global) average patient with the following characteristics:

Older than 60, male, short of breath for quite some time, family history, already visited multiple specialists, slow progressive onset, no other health problems in the past, smoker or remote smoker.

(Report 6)

With IPF considered to have the following impacts:

Not able to walk long distances any more, doing everything but slowly, stop hobbies, steps/slopes are avoided, lifting or carrying things/grandchildren, housework is very exhausting, showering and washing hair, needs help cleaning the house, cannot work anymore, no (long) vacations anymore, not able to fly anymore.

(Report 6)

No analytical explanation is provided as to how the construction was reached, nor which symptoms or impacts are prevalent in which of the people interviewed (e.g., by gender, age or ethnic subgroup).

In Report 3, 18 patients with IPF were interviewed from four countries, with the sampling criteria for patients being those on antifibrotic medication. In this report, the findings were presented per country (or market). The report acknowledges that at the time of the interviews, the United Kingdom was unique in restricting antifibrotic medication to patients with an FVC of less than 80% (NICE), and by combining interview data from only two UK patients, this leads to a perception of

UK patients acceptance of their lot in life: In the UK there is less worry expressed with the prognosis of IPF. Patients reach a cold acceptance with an untimely death. This attitude translates to less motivation to seek new treatments or adjustment in their lifestyle.

(Report 3)

Again though, no methodological detail was provided as to how this construction of a UK persona was reached from two interviews.

Patients were completely absent in the TPP report that focused on the uptake of a future new medicine for IPF. Physicians were presented with three hypothetical drug profiles (moonshot, mid and minimal drug profiles), with the most speculative (moonshot) proffering improved efficacy and improved quality of life. UK physicians were enthusiastic and liked the ‘promise’ of the (imaginary) drug; although it was not clear how the restrictions on prescribing antifibrotics may have informed these opinions, and no implications for patients (e.g., treatment regimens or side effects) were provided:

I like the fact that the important thing is that it reversed FVC decline, which is this key thing.

—Pulmonologist, UK(Report 5)

It sounds quite promising. The safety and tolerability is better and it doesn't mention any specific side effects.

*—*Pulmonologist, UK(Report 5)

Information was not provided about how these profiles were explained to the participating physicians (e.g., how the information was constructed, nor the thresholds for the three analytical categories (e.g., similar/different) (Report 5). Here, ‘end users’ were perceived as prescribers, rather than people with IPF.

### 3.2 Empirical Research

Interviews were conducted with patients with IPF, who often had partners and caregivers present in the interviews (Table 4). Interviews with staff were generally conducted when they were present in the specialist ILD centre, or their usual place of work (Table 5).

**Table 4:** Patient sample.

‘Trial/study’ experience (*n* = 12)	‘Non‐trial’ experience (*n* = 20)
*Location* County 1 = 9 County 2 = 2 Other = 1 (County 3)	*Location* County 1 = 10 County 2 = 6 Other = 4 (County 4, County 5, County 5, County 6)
*Gender* Male = 10 Female = 2	*Gender* Male = 15 Female = 5
*Age* Mean 72.2 Range (58–85)	*Age* Mean 72.2 Range (58–95)
*Deprivation Index Decile* Mean = 5.5 Range (3–9)	*Deprivation Index Decile* Mean = 5.5 Range (3–9) Missing = 3

**Table 5:** Clinical and trial staff sample.

Clinical staff (*n* = 12)	Trial staff (*n* = 7)
Consultant physician (1) General practitioner (2) ILD nurse (2) ILD support workers (2) Lead/specialist nurse (2) Pharmacist (1) Physiotherapist (1) Physiologist (1)	ILD registrar (1) IPF fellow (2) Pharmaceutical representative (1) PhD student (1) Research nurse (1) Research manager (1)
Location County 1 = 12	Location County 2 = 5 Other = 2 (Region 1, Region 2)

Abbreviation: ILD = interstitial lung disease.

Interviews were conducted with older men and women with multiple comorbidities, with experience of having an IPF diagnosis from 6 months to 12 years, living in areas that reflected a range of incomes and access to resources (such as community hospitals and transport hubs). Reflecting the demographics of the regional population (where 95% of the population identify as white [35]), 30 were white British, with two from a mixed ethnic background. Patients had a range of experiences of participation in clinical trials (Table 6).

**Table 6:** Patients understanding of trial participation.

Previous trial participants *n* = 12	Previous non‐trial participants *n* = 20
P1: 72, male, South County 1 (6) Observation 1 Trial 1 Trial 2	P2: 58, male, West County 2(4) Trial 10 Expressed interest, but did not participate Trial 11 Expressed interest, but did not participate
P3: 85, male moved to North England (5) Observation 1 ‘Trial’ of a medicine	P5 71, male, North County 2 (6)
	P6: 72, male, South County 2 (4)
P4: 72, female, Central County 1 (7) Audit 1 Trial 1 Trial 4	P10: 77, male, moved to East England (5) Thinks that he was in a trial at ILD centre
	P11: 75, male, West Cornwall (6) Expressed interest in a trial but did not meet inclusion criteria (comorbidity)
P7: 79, male, Central County 1 (6) Trial 1 Patient was recruited but did not start due to administrative error	P13: 76, male, East County 1 (8) ‘Trial’ of a medicine
P8: 82, male, South County 2 (3) Trial 5	P14: 59, female, Central County 1 (4) ‘Trial’ of a medicine Expressed interest in a trial but did not meet inclusion criteria (thinks she was eligible)
P9: 84, male, South County 1 (4) Observation 1	P20: 69, female, West County 1 (3)
P12: 62, male, Central County 1 (5) Trial 6 Trial 7 Patient did not recall study	P21: 82, male, West County 1 (6) Has ‘put his name down for research’
P15: 58, male, North County 2 (4) Trial 5 Trial 8	P22: 75, male, North County 1 (5)
	P23: 72, male, Southern England (6) Made enquiries directly to scientist re: PRE_CLINICAL_TRIAL1
P16: 81, male, South County 1 (6) Trial 5	P24: 71, male, Bristol (8) Provided with information about Audit 1
P17: 72, female, South County 1 (7) Trial 3 Trial 5	P25: 75, male, South County 1 (5)
	P26: 65, male, North County 1 (not known)
P18: 69, male, East County 1 (4) Trial 5	P27: 71, female, Mid County 2 (7)
P19: 69, male, South County 1 (9) Observation 1 Patient did not recall study TRIAL9	P28: 78, female, East County 1 (9)
	P29: 76, female, North County 1 (not known)
	P30: 62, male, West County 2 (6)
	P31: 76, male, East County 1 (9) ‘Trial’ of a medicine
	P32: male (95) Carer interviewed as patient in care home in Central England

*Note:* Trials 1–12 = trials as discussed by patients; Observation = observational study; number in brackets (*n*) = Deprivation Index Decile—Not known where postcode not recognised.

Patients' clinical and trial experiences were underpinned by an array of understandings of clinical trials and the potential for a stake in trial design, which clinical and trial staff worked hard to identify, interpret and inform (Table 7).

**Table 7:** Empirical data: Results (examples).

		Patient perspectives	Clinical and trial staff perspectives
Trial information	Information provision	‘On the whole, I've been pretty pleased with how it's been explained to me, whether through the respiratory specialist. It's been well explained.’ (P12) ‘No, you don't always remember the information, do you? With seventeen pages, by the time you've got to the end you've forgotten.’ (P20's husband) ‘You know, it's almost I feel that there seems to be too much concern with privacy on these things… I had to fill in various forms for them to actually be able to contact me to ask me if I wanted to do it sort of thing, which… well, why do you think I filled in the bloody forms?’ (P19)	‘Patients get thrown numbers. They don't really know what they mean’ (C6) ‘[Information sheets] are pages and pages long. They are not accessible. The average reading age of our population is 9 to 11 years old’ (T5) ‘I [try] to individualise the taking part in the study and finding out which part of taking part in a study will click for that patient. Because they're all… everyone's going to have different preferences for why they want to take part in research… I think we need to change the mindset of who we are recruiting into these studies. We should be recruiting the, quote unquote, average patient that comes through our door, not the perfect patient that comes through the doors… Until we have RCT evidence, we might actually be harming them’ (T7)
	Decision to participate	‘If I had sickness and diarrhoea every day, I think I would still take it… Because it's a lifeline. It's my lifeline, it's my only chance of living to an older age.’ (P14) ‘I wanted to do it anyway [regardless of the information] because I thought if it's research, then they need to know… they need to have people like guinea pigs to help them do this research because they're trying to help people, so if they're doing that, the least you can do is go along and help them to research the drug and get a study up and running.’ (P4)	‘The main reason I get from people [for trial non‐participation] is, “I just don't want to take another tablet if it's not going to make me feel better”….I've [also] had loads of conversations where it's, “I just want something”, “I just want anything”’ (C7) ‘Generally, it's someone who is engaged in the process…engaged in their condition… if the patients are really unwell, in the last stage of their journey…a study is unlikely to be something that they will want to do’ (T4)
Trial participation	Trial practices	‘I was put on a trial about 2 years ago, and it was about to start, [drug] trial, and the nurse didn't actually tell the [drug] people that I was starting…it all became a bit embarrassing, because I hadn't been put on the [drug company's] list.’ (P7) ‘[Randomisation] was mentally challenging…I would be disappointed if turned out I wasn't on it and didn't benefit’ (P6) ‘I saw that [ILD centre elsewhere] were doing trials for IPF… [Physician] said: “We don't think you're suitable for the trial we've got planned, but what we can do, we can…we can put you on Nintedanib and that will help the situation”… Down here in [County] I couldn't get Nintedanib.’ (P15) ‘[Physicians] phoned me and said we've got to stop [trial drug] because… he told me they had trouble… one of the patients on [drug] became unwell…They did a full check on me and everything was ok’ (P1) ‘It was just over a year and then [trial] was stopped…I was hoping I would find out more why it was stopped, but, well I didn't get any feedback on that’ [P1] ‘[When trial was stopped] I was devastated because I thought it was doing me some good… I was back at square one’ (P3)	‘[Patients are] looking for the opportunity to access new drugs that might work…regular contact with health staff outside of the standard clinical teams…emotional support…they're hoping that this might be the treatment that will…prolong life…and help them enjoy a better quality of life…it's just the reality of a system under pressure’ (T5) ‘In as much as we can see a patient and say, “There's a trial for XYZ, are you interested?” and they'll say yes, and then we will ask the research nurses to contact them about the trial and then send them some information on it. It can sometimes feel like weeks later, we are still no further along. I'm coming to appreciate a lot of that process is just making sure the boxes are ticked and the research nurses are going through, have they had a CT within 12 months, which might be for a clinical reason or for a study, and I think it's unfair to call it bureaucracy, but it feels like it's just working through the bureaucracy of getting a patient in, and it can be weeks down the line, and you feel like you haven't got anywhere…. Then there are questions about where are patients going to be prioritised for a CT scan, because people could be waiting a long time for a CT scan, and in terms of the resource allocation… So, lots of questions which seem quite bureaucratic to me, that can take a while to sort out’ (T4)
	Travel	‘I got a little allowance for the car if I went in the car. If I went on the bus, I got my money back, and before I had to have [medication], I had to go and have a donut, unfortunately.’ (P13) ‘I drove, and we stayed the night in a hotel [near ILD centre]. We're very lucky we have a big house and a big garden and [ILD centre] isn't out of reach’ (P2) ‘Rather than having to go 25 miles into [local hospital], it's probably 150 mile round trip to [regional centre]… it takes a whole day out of my time…I don't want to undergo any trials where I have to travel.’ (P8)	‘For the vast majority it's the time rather than being worried about the drug…I think the first thing people tend to think about is the getting back and forth from [ILD centre] and the time that involves… where we are in the geographical location is tricky, because it's really big, and not only is it really big, it's not got good transport networks. If it was [City], just a big area that had loads and loads of trains, that's a very different kettle of fish, isn't it? But, a lot of our patients are pretty isolated, to be honest, and making sure that just because you're isolated doesn't mean you're not represented is something that's probably very important.’ (T3)
	Hybrid participation	‘[Home spirometer] is a wonderful device… you get sort of home printed graphs like that, and the data can be sent to the hospital in a form that's comparable. But the book of instructions with it is absolutely useless and you have to actually remember how to do it each time.’ (P11) ‘Since I've been on the trial… I actually doubt sometimes, you know, whether or not the [home spirometry] information is getting through because there is no feedback to say even that yes, we've got the information that your spirometer has recorded.’ (P19)	‘[Trial participation] has to be individualised…I have just started a clinic at [primary care], so the patients at [rural location], they really struggle to come up to [local hospital], it's a long, long journey for them. So, I've got a nurse that works at [primary care]; she doesn't do ILD, but she might do a [blood] gas for me, or if there are issues with oxygen she may go and visit them at home, and then having the clinic at [primary care] reduces their journey time by about half an hour each way…it's just not feasible for me to be going out to [primary care] all the time; it's just not good use of my time.’ (C2) ‘Geographically, if they are such a long way away… you almost want something like a pop‐up clinic or like a pop‐up centre maybe… it's difficult, because there are some people that don't… technology is not… especially a lot of our patients are potentially older, then it's not appropriate for everyone, is it’ (C3)
Participation in trial design	Engagement with patient organisations	‘I stopped looking at [PO] website…it was amazingly depressing…I got fed up with reading about people gaining their “angel wings”!’ (P11) ‘We went to a [PO] meeting, we shan't go again…all they wanted was Nintedanib, when they weren't far enough forward to have it’ (P13) ‘I went a few times, but I think the benefits were outweighed by the fact I was there with a lot of other people that were dying, and looking at it that way, I'd prefer not to go.’ (P4)	‘There's an IPF support group…if you look at the demographic of the people on that, they're very academic, they're people that are very well researched, and the normal lay person finds it quite a difficult platform, just from feedback from some patients.’ (C6) ‘Patient representation groups, by nature, all the people who have the mental capacity, in terms of space in your bucket, to be able to do that. They are well enough to do that. Educated enough to do that.’ (T3)
	Engagement with pharmaceutical organisations	‘I don't think I'm clever enough to be helpful [to a drug company]…I'm not a team person!’ (P7) ‘They've got to talk to patients, whether they like it or not. [They] won't learn anything about the disease, unless they talk to patients like *me*’ (P5) ‘The companies can produce a drug, but unless they know how a patient feels, they don't get the full story… go to as many people as they can’ (P15)	‘You go to [pharmaceutical events] and you have your star patient. And your star patient is the one that gives an opinion on all these things… Trying to reduce the burden of being involved with patient participation, because obviously a lot of these things require time, require travel, require, you know, even access to Teams to be able to discuss with people. And I think that is probably a big barrier for a lot of patients to get involved with designing research studies’ (T7)

Abbreviations: C, clinical staff; P, patient; T, trial staff.

#### 3.2.1 Information and Understanding

Although patients were provided with information about available clinical trials, this was often considered confusing, because of the amount of information provided,^2^ as well as the conflation of information about a clinical trial with information about IPF, as often both were provided in the first clinic appointment at the ILD centre, as staff were mindful of the distance that patients had travelled and the length of time that they had waited for support:

I have recently, a couple of patients who are under the impression that we are talking to them, just the clinical physician appointment, is talking to them about clinical trials… People's understanding of who we are, why they're here and what the disease is, is really variable when they come and see us. Some people have virtually no understanding of it and then some people are really clued up, they've already read all of the information, they know all about the drugs, and if they are coming from that perspective, then what we're telling them is obviously much less, whereas you're almost having to do a diagnosis discussion with them and then we're talking about drugs with potential complex side effects, and then we're talking to them about clinical trials; that's a lot. (T3)

This led to confusion around ‘trying’ one of two already licensed medicines for IPF, which could only be prescribed by an ILD specialist, or participation in a ‘trial’ of an experimental medicine, managed on behalf of a pharmaceutical company at an ILD centre:

Well I was given the choice of going on two tablets for the trial. [Nintedanib or Pirfenidone] They didn't suggest which one, I asked which one they would suggest, but they said, ‘This has got to be up to you’, so the other one might have been Pirfenidone.

(P3)

#### 3.2.2 Travel and Technology

Similar to accounts in the patient insight reports, most patients with IPF valued the support that was available at the ILD centre, often because of the difficulties already experienced in accessing referral and diagnosis. However, some expressed frustration at lengthy trial processes, which required time spent at the centre, but which ultimately could lead to their exclusion from a trial:

It was [Trial 3], and I was called into [ILD centre] for tests to see if I was suitable. So, I spent the best part of a whole day up there having blood tests and ECGs, I had blood taken off me and loads and loads of questions, so I did all that, then I had to wait for the scan. It took so long for the scan to be arranged that by the time it was done, all the tests became irrelevant, they were too old… Then I had to go back for another set of tests. So, I went back again and had the blood, the ECG, all the same things done again, only to be told at the end that I wasn't suitable… If they'd seen my first scan, they shouldn't have even considered me. I wasn't suitable at the end of the day, but I felt a lot of time and money had been wasted.

(P17)

Some patients were clear that they would rather not travel to the ILD centre to take part in a trial, either due to the distance or duration of the journey, or due to the extent of their IPF symptoms and/or other health conditions:

What I wouldn't want to do is have to travel. Going to [local hospital] is bad enough, I'm afraid… It's probably a hundred‐ and fifty‐mile round trip to [ILD centre]. I don't want to go to [ILD centre] at all.…

(P8)

These issues were long recognised by the staff who support them or who are required to assess their suitability, with all mentioning the need for an effective hub and spoke model of service delivery, as well as the challenges of technological solutions to enable hybrid trial delivery, which were viewed as challenging for older people and/or those perceived of as lacking in technological know‐how:

I don't know if there would be middle ground whereby, rather than everything being totally remote or totally in [ILD centre], or in whatever centre it may be, whether there'd be a middle ground, whereby we'd have satellite teams in other hospitals around who could do what we do in [ILD centre]. That might bridge that gap.

(T4)

People would be much more willing [to participate in trials] if they could do things from home … [but] our group of patients are older and therefore their digital fluency is much less. We've had the trial with the home spirometry and some of the problems we found with that is that some people don't have a smartphone, if they do, they don't know how to use it, they may or may not have internet.

(T3)

#### 3.2.3 Opportunities to Engage in Trial Design

Patients interviewed identified themselves as different from patients who attend patient organisations (from where patients are typically recruited for patient insight reports), who they typified as either evangelical about new drugs, or miserable once they are too sick to participate in trials:

We went to a [patient organisation] meeting, we shan't go again…all they wanted was Nintedanib, when they weren't far enough forward to have it.

(P13)

I went a few times, but I think the benefits were outweighed by the fact I was there with a lot of other people that were dying, and looking at it that way, I'd prefer not to go.

(P4)

This is important as pharmaceutical companies undertake patient engagement with disease‐specific or umbrella patient organisations, rather than individual patients. Furthermore, the patients that were interviewed suggested that they were not ‘smart enough’ to take part in research, or they could not imagine mechanisms that would enable them to work with drug companies on an even footing. Without understanding the value of their contribution, some thought it was best left to doctors to advocate on their behalf:

I don't think I'm clever enough to be helpful [to a drug company]…I'm not a team person!

(P7)

I have no view at all. I'm not really qualified. I'll have whatever's going. If it helps, well fine, I'd rather have the one that's going to make a difference than have a placebo, but there you go.

(P2)

Thus, patients in the patient insight reports lost their ‘voice’ due to methodological practices that simplify their experiences for a time‐sensitive industry audience, whereas those interviewed lost their stake in patient engagement because they could not imagine how things could be otherwise.

## 4 Discussion

Our analysis of patient insight reports identified how the perspectives of some patients with IPF are excluded from the evidence‐base that informs trials of new medicines for the disease (and subsequent marketing of products), by the methodological practices that construct them. This finding was amplified by those from the empirical interviews which showed the complexity of information given to patients, the burden of trial participation and the absence of opportunities to engage in trial design further negate some patient voices [36, 37, 38].

A recent Reflections Paper produced by the ICH [12] acknowledges that the current practice of collecting patient insights has significant room for improvement. The paper identifies ‘key areas where incorporation of the patient's perspective could improve the quality, relevance, safety and efficiency of drug development and inform regulatory decision making’. However, having ‘reflected’ the paper notes that the incorporation of patient perspectives in the drug development lifecycle is indeed a challenge, which now requires further stakeholder consultation [12].

Previous research has identified that the appropriate and innovative use of codesigned qualitative research and patient and public involvement and engagement has the potential to capture the perspectives of under‐researched, underserved and seldom‐heard communities, which could realign the research agenda to include those who are currently absent [18]. By employing this approach, we have here been able to identify where patients with IPF currently ‘get lost’ in research and suggest research practices which might enable things to be different.

Rabeharisoa et al. [39] have written about the ‘politics of numbers’ in rare disease research, whereby patients pool their knowledge and political resources (e.g., in patient organisations) to engage pharmaceutical organisations, to meet their challenges and unmet needs. By doing so, they provide a ready sample of patients for recruitment by third‐party vendors to patient insight exercises, but this reification means that other demographic variables become redundant, such that the third‐party vendors only need to sample a given number of patients with a condition because that is the only variable of interest. Therefore, other patient characteristics are lost as they become the representative of the condition [40]. A corollary is that patients become one‐dimensional, and the reports often constructed an average patient, rather than a more multidimensional person. Thus, patients with IPF are represented as male, frail and likely to have met many specialists before being diagnosed, and this ‘patient persona’ becomes the standard for the population [15, 41].

Zvonareva [42] notes that because business is premised upon efficiency, it is unsurprising that the tools are standardised to optimise their re‐use and scalability. But, although such tools might optimise the return on the time invested in an interview, they limit the extent to which a patient can raise and discuss the issues that are more important to them, but which fall outside of the remit of the research brief [43, 44].

We noted that where patients from multiple markets were engaged, the results were presented in that format (as the unit of business), which could lead to slippage into cultural stereotypes (e.g., the cynical or stoic UK citizen, the free market favouring American and the heavily smoking Italian), rather than going beyond common‐sense understandings, and adding value to our understanding [45].

Brown and Michael [46] identified that expectations for a technology are mediated by an actors' involvement and proximity to the technology. To inform the Total Product Profile (which for proprietary interests cannot be reported here in full), it is the physicians closest to the innovation, by virtue of being the specialist physicians with an established relationship with a pharmaceutical company, or who have been involved in clinical trials of near‐market products who are recruited. In the reviewed report, they supported the ‘moonshot’ product with the most speculative and ambitious profile (the most disruptive), rather than the ‘minimal scenario’, which is more likely to provide a small incremental (and less risky) scenario for patients [47].

This research has demonstrated how narrow opportunities are for people with IPF to engage in research to meet their needs. Patient and professional experiences and needs are constructed by commercial interests, such that some patients currently lack engagement in both trial design and trial practices [48]. These practices obfuscate wider patient perspectives, in a way that continues to put the needs of business, or patients with existing relationships with business, before those of other patients—limiting the potential for the development of personalised medicines [49]. Change in methodological practices would enable wider patient engagement [50, 51]; but to do so would require new knowledge production relationships and professional relationships, particularly upstream in the drug development lifecycle [52]. Although current practice exacerbates existing inequalities, opportunities for recalibration will only be possible when pharmaceutical organisations strategically employ mechanisms for meaningful representation and inclusion [53].

A limitation of this research is that not all methodological details for the patient insight reports were available for appraisal, due to their proprietary nature. However, a strength is that in the spirit of the knowledge transfer partnership, the anonymised content of the reports was made available for discussion by patients, clinicians and pharmaceutical representatives, thus preventing research waste [54] and enabling the opportunities for innovation to be identified.

## 5 Conclusion

People with IPF want the opportunity to participate in drug trials and trial science. We undertook empirical research, guided by the concerns of our patient collaborators with personal experience of IPF, and which included IPF patients with a range of demographic characteristics. Patient participants identified that they have a range of needs that can inhibit their engagement, but which they think that pharmaceutical companies need to do more to accommodate. Similarly, staff at the interface of trial science identified the work that they needed to do to support patients' decision making, but some see current practices as suboptimal for personalising information and making trials accessible. Through critical appraisal of patient insight reports we identified that current industry practice ensures that some people are engaged, whereas others are not. Patients were typically only sampled from patient organisations, which patients in our empirical study did not engage with; while the clinical staff who were sampled were more likely to be enthusiastic about the most innovative and potentially risky new therapies—rather than those which a wider group of patients might be able to access and adhere to. We propose that methodological rigour (e.g., sampling for equality, diversity and inclusivity) and deliberative practices (e.g., developing drug trial research with rather than on patients) are required to enable a wider group of patients to have a stake in the design of drug trials for IPF and access to new treatments. The challenge now is for regulators to mandate such inclusive practices and for the pharmaceutical industry to adopt them.

## Author Contributions

**Julia Frost:** conceptualization, investigation, funding acquisition, methodology, validation, visualization, software, formal analysis, data curation, writing – original draft, writing – review and editing, supervision, project administration, resources. **Jessica Mandizha:** writing – review and editing, methodology, data curation, supervision, investigation. **Steve Jones:** conceptualization, investigation, funding acquisition, writing – review and editing, formal analysis, supervision, data curation. **Chantal van den Dungen:** investigation, writing – review and editing, supervision, data curation, formal analysis. **Lauren Asare:** project administration, writing – review and editing. **Catherine Pope:** conceptualization, investigation, funding acquisition, writing – review and editing, methodology, validation, formal analysis, data curation, writing – original draft.

## Widening Engagement Patient Advisory Group

Health and Community Sciences, University of Exeter Medical School, University of Exeter, South Cloisters, St Luke's Campus, Exeter, EX1 2LU. WideningEngagementPAG@exeter.ac.uk.

## Ethics Statement

The study was approved by the London ‐ Riverside Research Ethics Committee (REC reference: 22/LO/0186; IRAS project ID: 304904).

## Consent

All participants gave their written consent for the interview in which they participated to be recorded and declared that they understood that the interview would be typed up (transcribed), with all of the information anonymised.

## Conflicts of Interest

The authors declare no conflicts of interest.

## Data Availability

Transcripts will not be shared in their entirety to protect the anonymity of patients and health care professionals. However, requests for excerpts of the data will be considered on an individual basis. Please contact the corresponding author.

## Endnotes

1 Interviews with patients were conducted before the change in NICE [55] guidance, which extended the use of Nintedanib for patients with idiopathic pulmonary fibrosis (IPF) with an FVC of above 80%. This change was implemented while interviews with staff were in progress. This change effectively widened access to antifibrotic therapies in line with that reported in the patient insight reports, but in the short term may add an additional 5000 patients to the ILD workload (NICE [56]).

2 Understanding research information is difficult for some people. Of the 130 people with IPF approached to take part in this interview study, 21 completed the consent form incorrectly and thus could not be approached to participate, while three of those interviewed asked if the qualitative interviewer was recruiting them for a clinical trial.
